# Deep Learning-Based Magnetic Resonance Imaging Features in Diagnosis of Perianal Abscess and Fistula Formation

**DOI:** 10.1155/2021/9066128

**Published:** 2021-10-22

**Authors:** Jun Yang, Song Han, Jihua Xu

**Affiliations:** Department of Anorectal Surgery, Qilu Hospital (Qingdao), Cheeloo College of Medicine, Shandong University, 758 Hefei Road, Qingdao, Shandong 266035, China

## Abstract

There was an investigation of the diagnostic and prognostic effect of magnetic resonance imaging (MRI) based on multimodal feature fusion algorithm for impotence of perianal abscess. In this study, the second to fifth convolution blocks of the visual geometric group network were applied to extract the depth features in the way of transfer learning, and a multimode feature fusion algorithm was constructed. The whole network was trained by maximizing the energy proportion of the feature layers, which was compared with the fully convolutional neural network (FCN) algorithm. Then, this algorithm was adopted to the imaging diagnosis of 50 patients with anorectal diseases admitted to our hospital, and it was found that the similarity coefficient (85.37%), accuracy (80.02%), and recall rate (79.38%) of the improved deep learning algorithm were higher markedly than those of the FCN algorithm (70.18%, 67.82%, and 66.92%) (*P* < 0.05). As the number of convolutional layers increased, the segmentation accuracy of the convolutional neural network (CNN) algorithm was also improved. The detection rate of the observation group (84%) rose hugely compared with the control group (64%), and the difference was statistically obvious (*P* < 0.05). Besides, the detection accuracy of abscess location (84%), impotent tract location (80%), and internal orifice location (92%) in patients from the observation group was higher substantially than the accuracy of abscess location (60%), impotent tract location (68%), and internal orifice location (72%) from the control group (*P* < 0.05). In conclusion, the performance of the multimodal feature fusion algorithm was better, and the MRI image feature analysis based on this algorithm had a higher diagnostic accuracy, which had a positive effect on improving the detection rate, detection accuracy, and disease classification.

## 1. Introduction

Perianal abscess is a very common perianal disease, which is called anal pain and dirty poison in traditional Chinese medicine. The incidence rate is increasing every year, and it is more common in young men [1]. Currently, perianal abscesses are mostly thought to be caused by metastatic gland infections. Human anal glands have a flask-like shape, composed of glands and ducts, with 6–10, which are used to secrete acidic mucus to lubricate the anal canal [2, 3]. Perianal abscess is caused by infection of the anal gland located between the sphincter muscles. The ways of infection include blockage of the anal glands, inability to drain the secretion of the anal glands, fluid accumulation in the glands, infection of the anal glands (between the sphincter muscles), the formation of swelling (infection source), and spread along with the conjoint longitudinal muscle to the perianal space [4]. The diagnosis of perianal abscess and anal canal stenosis is clear before surgery, and the location of the perianal opening, abscess range, anal fistula trend, and its relationship with surrounding muscle tissue are clearly defined, which is of great significance for the selection of surgical methods and the protection of anal physiological functions [5,6]. The recurrence rate of the anal canal is high, mainly because the loose canal is missed during the surgery or the occult abscess cannot be diagnosed before the surgery. The treatment of such patients is very difficult, and the key to solving this problem lies in accurate diagnosis before surgery [7].

In recent years, with the rapid development of magnetic resonance imaging (MRI) technology, nonionizing radiation MR soft tissue has high resolution and can directly image three-dimensionally. Due to the low movement of pelvic organs, high-quality images can be collected, which can accurately describe the anatomical structures of the internal and external anal sphincter, levator ani, and puborectalis muscles and can show the relationship among perianal abscess, anal fistula, and muscles around the anus [8]. People have gradually realized that the application of preoperative MRI has affected the choice of treatment methods for anal impotence, especially for the treatment of recurrent anal impotence [9]. At present, MRI has been used by scholars in developed countries as the gold standard for the assessment and classification of anal fistula. However, there are no detailed reports in domestic and foreign literature for the axial coronal sagittal plain scan and enhanced sequence in the scan, which type of internal opening of the failing tube is easy to display, and which type of internal opening of the failing tube is easy to miss the diagnosis. Preoperative MRI examination of the perianal abscess has not attracted much attention. At present, deep learning methods have surpassed traditional medical image analysis methods and made great progress in the field of anorectal MRI of patients. Lv et al. [10] developed an anorectal MR injury detection system based on deep learning. The system consists of two convolutional neural networks (CNNs): the first CNN network is used for fast image segmentation; the second CNN network is adopted to evaluate structural abnormalities of the anorectal tract.

Based on the above, the objective of this study was to explore different MRI sequence imaging based on the deep learning algorithm, detect different types of perianal abscess and anal fistula, and conduct a detailed statistical comparison with surgical results to determine the best-combined scanning sequence. Therefore, it could help surgeons make accurate surgical plans before surgery for perianal swelling and anal fistula and minimize the damage caused by surgery to patients, which played a very critical role in postoperative recovery and preservation of defecation function of patients. It was hoped that, to some extent, this research could provide a reference for the clinical diagnosis of perianal abscess as impotence disease, thereby providing some new ideas for the diagnosis of the disease.

## 2. Materials and Methods

### 2.1. Selection of Research Objects

In this study, 50 patients with anorectal diseases were selected as the research objects, who were admitted to the hospital from January 20, 2018, to February 15, 2020. Then, all of them were grouped randomly into the observation group and the control group. The observation group was diagnosed by MRI based on the deep learning algorithm, while the control group was diagnosed by routine MRI. Finally, the postoperative pathological results were compared and analyzed with the above results. This study was approved by the Medical Ethics Committee of the hospital, and the patients and their family members understood the situation of this study and signed the informed consent forms.

### 2.2. Inclusion and Exclusion Criteria

The criteria for inclusion were defined to include patients who were in this hospital, met the diagnostic criteria for anal fistula, and underwent surgical treatment in the anorectal department of this hospital; had the true and reliable cases; agreed to cooperate with clinical investigators.

The diagnostic criteria for anal fistula are as follows according to the *Standards for the Diagnosis and Treatment of Anorectal Diseases of Traditional Chinese Medicine Industry of the People's Republic of China* [11].

Pus discharge: local intermittent or continuous discharge of pus and the wound does not heal for a long time. Generally speaking, the initial formation of fistula has more pus, it is accompanied by fecal smell, and the color is yellow and viscous; after a long time, the pus is scarce or sometimes absent, showing intermittent pus; if the patient is too tired, pus will increase, and serious people even have feces outflow; if the pus is reduced and then suddenly increased and accompanied by anal pain, it may be considered as an acute infection or the formation of new branches.

Pain: when the fistula is open, there is usually no pain, but only a local swelling. If the external mouth closes on its own and pus accumulates, there may be local pain or cold and heat; if the purulent water flows out after ulceration, the symptoms can be rapidly alleviated or disappeared. However, the stool flows into the pipeline and causes pain because the inner mouth is larger, especially the pain is aggravated when defecating. The pus constantly stimulates the skin around the anus and causes itching, sometimes accompanied by perianal wet sores.

According to the unified classification standard of anal fistula formulated by the National Anorectal Collaborative Group held in Hengshui, Hebei, in 1975, the anal fistula can be divided into four categories, with the deep layer of the external sphincter as the boundary [12]. (1) Low-position simple anal fistula: the internal mouth is in the anal crypt, with only one tube and passing through the deep layer of the external sphincter. (2) Low-position complex anal fistula: the internal orifice is in the anal crypt, with only one tube passing through the deep layer of the external sphincter and two or more external orifices and channels. (3) High-position simple anal fistula: the internal mouth is in the anal recess, with only one tube, running above the deep layer of the external sphincter, invading the puborectalis or levator ani muscle. (4) High-position complex anal fistula: there are one or two internal openings, two or more ducts, and branch cavities. The main duct passes through the deep layer of the external sphincter and invades the puborectalis muscle or above the levator ani muscle.

The criteria for exclusion were defined to include patients who did not undergo surgical treatment in the anorectal department of this hospital; lost contact; and were unwilling to cooperate with clinical investigators.

**Figure 1:**
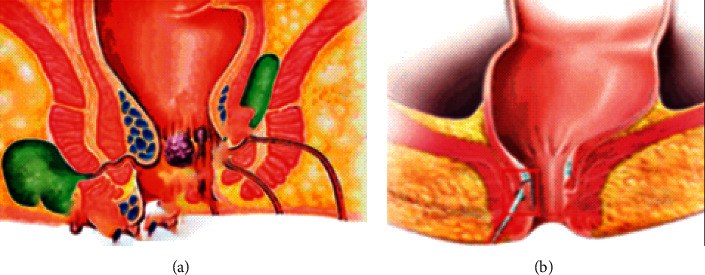
A perianal abscess (a). Anal fistula (b).

Figure 1 shows the perianal abscess and anal fistula.

### 2.3. Observation Indicators

Enhancement images of axial T1-weighted imaging (T1WI), T2-weighted imaging (T2WI), coronal T2WI, and coronal T1WI were analyzed by MRI. The patients were revisited through telephone outpatient service to record the formation rate of anal fistula, the incidence of anal keyhole malformation, and wound healing time.

### 2.4. MRI Examination

After the patient entered the imaging laboratory, the nursing staff guided him to take the lithotomy position, and the attending physician used the finger diagnosis method to evaluate the condition of the perianal lesions. Before the examination, the patient was guided to empty the rectum, take the supine position, and perform an MRI examination. The coil center was placed in the symphysis pubis center and scanning examination [13].

1. T1WI for fat suppression in transverse axis: time to repetition (TR) was 4,000 ms, time to echo (TE) was 80 ms, layer thickness/layer spacing was 4.0/1.0 mm, the field of view (FOV) was 280 × 280 mm, the matrix was 182 × 182, and the number of scanning layers was 15
2. Axial diffusion weighted imaging (DWI): *b* = 0, 800 s/mm^2^, TR = 5,000 ms, TE = 90 ms, layer thickness/layer spacing = 4.0/1.0 mm, FOV = 280 × 280 mm, matrix = 352 × 352, and 20 scanning layers
3. Sagittal T2WI: TR = 4,000 ms, TE = 80 ms, layer thickness/layer spacing = 4.0/1.0 mm, FOV = 280 × 280 mm, matrix = 182 × 182, and 15 scanning layers
4. T2WI of coronal fat suppression: TR = 4,000 ms, TE = 85 ms, layer thickness/layer spacing = 4.0/1.0 mm, FOV = 300 × 300 mm, matrix = 570 × 450, and 18 scanning layers
5. Axial T1WI: TR = 500 ms, TE = 12 ms, layer thickness/layer spacing = 4.0/1.0 mm, FOV = 260 × 260 mm, matrix = 450 × 450, and 20 scanning layers
6. LAVA-Flex dynamic enhanced scanning with breath-holding axis: TR = 5.1 ms, TE = 1.2 ms, layer thickness = 2.0 mm, FOV = 278 × 380 mm, matrix = 278 × 218, and 35 scanning layers

According to the actual situation of the patient, the contrast agent was injected, and the examination was carried out. The MRI reports and conclusions of all patients were reached after the unanimous opinions of two radiologists. All cases underwent surgical treatment within 1 week after the MRI examination. The specific details of abscesses and anal fistulas (internal opening, fistula, and external opening) were recorded, which were compared with the MRI information for analysis.

### 2.5. Construction of Deep Learning Model Based on Multimodal Feature Fusion

#### 2.5.1. Deep Feature Extraction

At present, CNN is more and more widely applied in the field of medical image diagnosis and has made good progress. The mechanism of CNN is to automatically complete the feature extraction of the image through the convolutional operation of the image, which has higher semantic information and stronger robustness [14]. Since the deep learning model can achieve better results only when it is trained on labeled images, the second to fifth convolution blocks of pretrained Visual Geometry Group Network 16 (VGG16) [15] are directly adopted in this study in the way of transfer learning. The feature map of the last layer of each convolution block is extracted and processed, with *H*(*g*) and *g*=1,2,3,4,5. *H*(*g*) is upsampled and *H*(*g* − 1) uses 2*∗*2 convolution to perform pixel fusion on the result image, so a 5*∗*5 convolution kernel is applied to correct the fused image, which can eliminate the aliasing effect used above and obtain a new feature map *H*(*g* − 1). Besides, the pyramid fusion equations are shown in the following equations:

(1) $$\begin{array}{c} K\left(g-1\right)=y_{2\times 2}\left(H\left(g-1\right)\right), \end{array}$$

(2) $$\begin{array}{c} H\left(g-1\right)=y_{5\times 5}\left(H\left(g\right)+K\left(g-1\right)\right). \end{array}$$

After *H*(2) of the last layer is obtained, it passes through the batch normalization (BN) layer, the adaptive maximum pooling layer, and the fully connected layer in turn. The BN layer can speed up the convergence speed and classification effect of the model. *y*^(*n*)^ is set as the *n*-th dimension feature of *H*(2); the BN layer is to introduce *y*^(*n*)^ into the parameters ∂^(*n*)^ and *ƛ*^(*n*)^, and the indifference estimation was employed to output the *n*-th dimension feature.

(3) $$\begin{array}{c} y^{\left(n\right)}=\partial^{\left(n\right)}{\overset{-}{s}}^{\left(n\right)}+{ƛ}^{\left(n\right)}. \end{array}$$

Here, ${\overset{-}{s}}_{l}=s_{l}-q/\sqrt{w_{x}^{2}+\vartheta },q=1/e\sum_{l=1}^{a}s_{l}$ is the average value of batch size *q* and *w*_*x*_^2^=1/*e*∑_*l*=1_^*a*^(*s*_*l*_ − *w*_*x*_) is the variance of batch size *q*.

When nonlinear factors are added to the ReLU layer, the expression ability of the increased model will be weakened. The activation function of ReLU is shown as follows:

(4) $$\begin{array}{c} f\left(m\right)=\max\left\{0,m\right\}. \end{array}$$

The main difference between the adaptive maximum pooling layer and the standard MaxPooling is that the former will control the output-size (Out) according to the input-size (In), stride, and kernel size which are displayed in equations (5)–(7).

(5) $$\begin{array}{c} \text{stride}=\text{floor}\left(\frac{\text{In}}{\text{Out}}\right), \end{array}$$

(6) $$\begin{array}{c} \text{kernelsize}=\text{In}-\left(\text{Out}-1\right)\times \text{stride,} \end{array}$$

(7) $$\begin{array}{c} \text{Padding}=0. \end{array}$$

The fully connected layer can be regarded as the full-scale convolution of *s* × *u*, *s* and *u* are the output size of the previous layer, and finally 1,026-dimensional features extracted by CNN can be obtained as follows:

(8) $$\begin{array}{c} h=\left(h_{1},h_{2},h_{3},\ldots ,h_{1026}\right). \end{array}$$

#### 2.5.2. Adaptive Fusion of Multimodal Features

Due to the different features of different modalities [16], a deep learning model of multimodal feature fusion was constructed in this research, to preserve the correlation of multimodality. The model contains a hidden layer whose number of neurons is less than the feature dimension and a sigmoid layer. The whole network is trained by maximizing the energy proportion of the feature layer. The sigmoid layer can map the feature interval after feature fusion to between (0,1), which is the prediction probability. Let the feature vector *m* = (*c*, *o*), and the forward propagation equations are as follows:

(9) $$\begin{array}{c} p_{e}=\left(s_{i}+t_{i}\right)\vartheta_{ie}+\beta_{e}, \end{array}$$

(10) $$\begin{array}{c} W=\partial \sum_{l=1}^{a}\left(s_{i}+t_{i}\right)\vartheta_{ie}+\beta_{e}. \end{array}$$

In equations (9) and (10), ∂(*k*)=(1/1+*n*^−*k*^), *t*_*i*_ is the deviation of the visible layer, *β*_*e*_ is the deviation of the hidden layer, and *p*_*e*_ is the hidden layer vector. To obtain the optimal fitting multimodal feature, the energy model is used to adjust the parameters, and the energy function is presented as follows:

(11) $$\begin{array}{c} H\left(x,y|\ell \right)=-\sum_{l=1}^{a}s_{i}t_{i}-\sum_{e=1}^{i}\beta_{e}p_{e}-\sum_{l=1}^{a}\sum_{e=1}^{i}t_{i}\vartheta_{ie}y_{i}. \end{array}$$

In equation (10), *ℓ*=(*t*_*i*_, *ϑ*_*ie*_, *y*_*i*_), *H*(*x*, *y|ℓ*) represents the total energy of the module.

The marginal probability distribution was defined, as follows:

(12) $$\begin{array}{c} p\left(x|\ell \right)=\frac{1}{Z\left(\ell \right)}\sum_{e}r^{-k\left(x,y|\ell \right)}, \end{array}$$

(13) $$\begin{array}{c} p\left(y|\ell \right)=\frac{1}{Z\left(\ell \right)}\sum_{i}r^{-k\left(x,y|\ell \right)}. \end{array}$$

In the above equations, $Z\left(\ell \right)=\sum_{il}r^{-k\left(x,y|\ell \right)}$ and the optimization function is defined, which can be displayed as follows:

(14) $$\begin{array}{c} \ell_{nm}=\arg\underset{\ell }{\max}\sum_{j=1}^{o}\mathrm{lg}P\left(x_{j}|\ell \right). \end{array}$$

**Figure 2:**
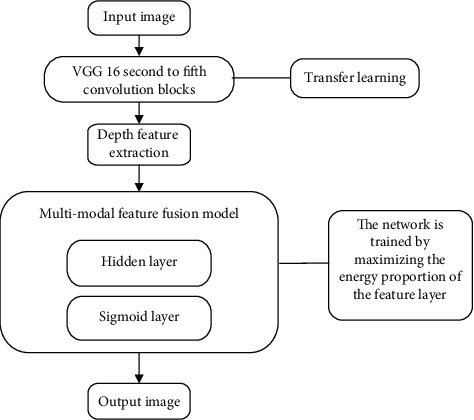
The algorithm flow chart of this paper.

Here, *o* is the number of samples. When function *ℓ* achieves the maximum value, the energy proportion of the characteristic layer is high, and the energy of the hidden layer is small. When transmitting data in the network, the direction of the data flow is also the direction of energy dissipation. After multiple iterations, the network energy shows a decaying trend, and the network tends to be ordered or the probability distribution tends to be concentrated [17]. The algorithm flow chart of this study is shown in Figure 2.

### 2.6. Evaluation Indicators

Accuracy, recall, and area under curve (AUC) values were adopted to quantitatively evaluate the performance of different models.

Accuracy is the proportion of correct samples predicted by the model to the total samples. The calculation equations are as follows.

(15) $$\begin{array}{c} \text{Accuracy}=\frac{\text{TP}+\text{TN}}{\text{TP}+\text{FN}+\text{TN}+\text{FP}}\text{,} \end{array}$$

(16) $$\begin{array}{c} \text{Recall}=\frac{\text{TP}}{\text{TP}+\text{FN}}\text{.} \end{array}$$

Here, true positive (TP) indicates that the segmentation result and the gold standard result are both true, that is, true positive; false positive (FP) means that the segmentation result is false, and the gold standard result is true; false negative (FN) indicates that the gold standard result is true, and the standard results are all false.

The AUC value is defined as the area under the receiver operating characteristic (ROC) curve enclosed by the coordinate axis. Since the ROC curve is generally above the line *y* = *x*, the value range of AUC is between 0.5 and 1. The closer the AUC is to 1.0, the higher the authenticity of the detection method.

### 2.7. Statistical Methods

The data processing in this study was analyzed by SPSS19.0 version statistical software. The measurement data were expressed as mean ± standard deviation (‾*x* ± *s*), and the count data were represented by percentage (%). The *t*-test and *χ*^2^-test were adopted for detection, and the correlation between different groups was for statistical analysis. The variance test was used between the groups, and the difference was considered statistically substantial with *P* < 0.05.

## 3. Results

### 3.1. Analysis of the Performance of MRI Images Processed by the Deep Learning Algorithm

**Figure 3:**
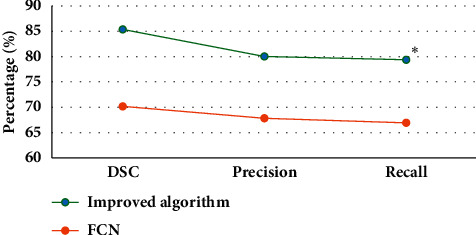
Comparison of the similarity coefficient, accuracy, and recall rate of the two algorithms (Note: *∗*indicated a statistically obvious difference compared with FCN algorithm (*P* < 0.05).).

The images processed by the deep learning algorithm based on multimodal feature fusion (improved algorithm) in this study were compared with the MRI images processed by the pure full CNN (FCN) algorithm under different training cycles.  Figure 3 shows that the similarity coefficient, accuracy, and recall rate of the improved algorithm were 85.37%, 80.02%, and 79.38%, respectively. The similarity coefficient, accuracy, and recall rate of FCN were 70.18%, 67.82%, and 66.92% in turn; the similarity coefficient, accuracy, and recall rate of the improved deep learning algorithm elevated hugely in contrast to those of the FCN algorithm, with a statistically marked difference (*P* < 0.05).

**Figure 4:**
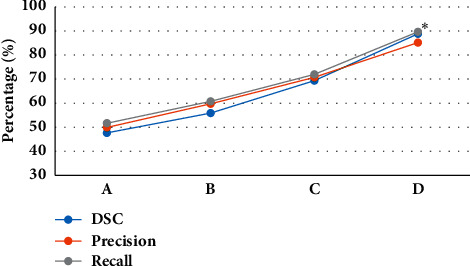
Comparison of the performance of different training layers (Note: A—the number of training layers was 6; B—the number of training layers was 10; C—the number of training layers was 15; D—the number of training layers was 20. *∗*indicated a statistically significant difference compared with the deep learning algorithm with 6 training layers (*P* < 0.05).).

**Figure 5:**
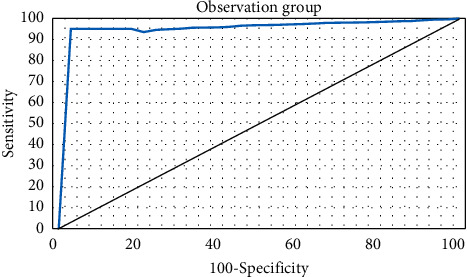
Comparison of the ROC curve of the observation group.

Figure 4 revealed that the similarity coefficient, accuracy, and recall rate of the deep learning algorithm with a total number of 20 layers were 88.82%, 85.19%, and 89.66%, respectively. The similarity coefficient, accuracy, and recall rate of CNN with 6 layers were 47.62%, 49.88%, and 51.62% in sequence. It was found that, with the increase of the number of convolutional layers, the segmentation accuracy of the CNN algorithm was also continuously improved. Compared with the deep learning algorithm of 6 convolutional layers, the segmentation accuracy of the deep learning algorithm of 20 convolutional layers was significantly improved, and there was a statistical difference between the two algorithms (*P* < 0.05). Furthermore, the AUC area of the observation group was 0.978, and that of the control group was 1.  Figure 5 shows the ROC curve of the observation group.

### 3.2. General Information of all the Patients

**Figure 6:**
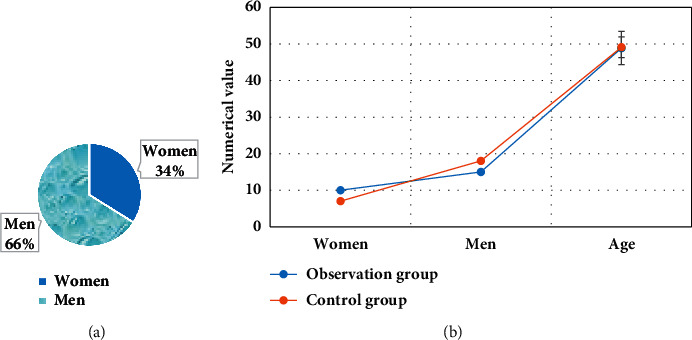
(a) Distribution of male and female gender ratios; (b) comparison on general information of patients from the two groups.

A total of 50 patients with anal abscess and atrophy were included in this study. There were 33 male patients and 17 female patients, ranging in age from 20 to 60 years old, with an average age of 48.19 ± 5.27 years old. There were 25 patients in the observation group, and 15 cases were male and 10 were female with an average age of 48.88 ± 4.56 years old; 25 patients in the control group had 18 males and 7 females, with an average age of 49.11 ± 2.82 years old. There was no statistical difference between the two groups, and they were comparable (*P* > 0.05) (Figure 6).

### 3.3. Displaying Results of MRI Images of all the Patients

**Figure 7:**
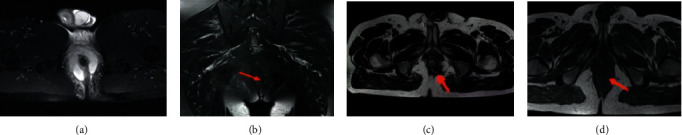
MRI images of patients with different types of perianal abscesses and fistulas. (a) Horseshoe fistula; (b) coronal T2WI fat suppression enhancement sequence; (c) T1WI sequence; (d) DWI sequence.

Figure 7 shows the MRI images of patients with different types of perianal abscess with impotence. Among them, Figure 7(a) is an image of horseshoe impotence, Figure 7(b) indicates the coronal T2WI lipo-inhibition enhanced sequenced linear sphincter impotence, Figure 7(c) is an image of the T1WI sequence uncomplicated trans-sphincter impotence, and Figure 7(d) is an image of the DWI sequence intrasphincter impotence with abscess.

**Figure 8:**
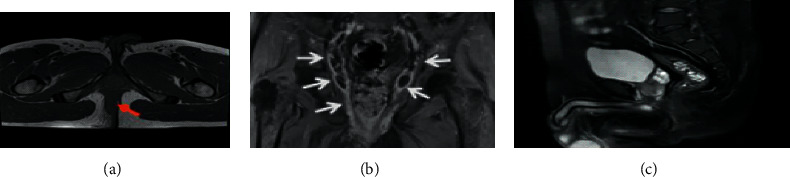
MRI images of a 48-year-old male patient. (a) Transverse axial enhanced scanning; (b) transverse axial T2WI sequence; (c) ultrasound images.

Figure 8 displays the 3 MRI images of a 48-year-old male patient. Furthermore, Figure 8(a) was a transverse axial enhanced scan of the image, showing impotence of the levator ani muscle; Figure 8(b) was the transverse axial T2WI sequence of the image, with complex anal impotence; Figure 8(c) was the ultrasound image to show that the fistula tube was <5 mm.

### 3.4. Comparison of the Detection Conditions of Patients from the Two Groups

**Figure 9:**
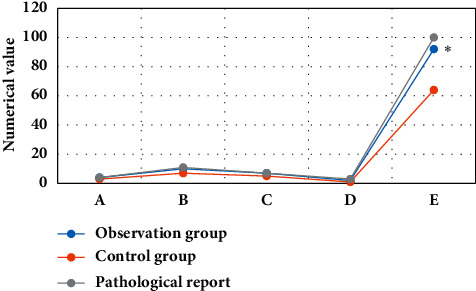
Comparison of abscess detection between the two groups. A—the intersphincter type; B—the trans-sphincter type; C—the suprasphincter type; D—the extrasphincteric type; E—the detection rate. *∗*indicated that the detection rate of the observation group was statistically different from that of the control group (*P* < 0.05).

After the postoperative pathological diagnosis of the patients with perianal abscesses, the results were as follows: 4 cases of intersphincter type, 11 cases of trans-sphincter type, 7 cases of suprasphincteric type, and 3 cases of extrasphincteric type. In the observation group, there were 4 cases of intersphincter type, 10 cases of trans-sphincter type, 7 cases of suprasphincteric type, and 2 cases of extrasphincteric type, with a detection rate of 84%. Furthermore, 3 cases of intersphincter type, 7 cases of trans-sphincter type, 5 cases of suprasphincter type, and 1 case of extrasphincteric shape were detected in the control group, and the detection rate was 64% (Figure 9), and the detection rate of the observation group was statistically different from that of the control group (*P* < 0.05).

### 3.5. Comparison of the Detection Accuracy of Patients from the Two Groups

**Figure 10:**
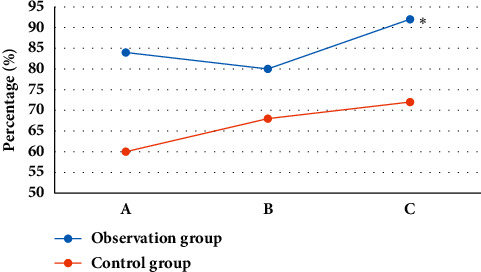
Comparison of the detection accuracy of patients from the two groups. A—the location of the perianal abscess; B—the location of the fistula; C—the location of the internal mouth. ^*∗*^indicated that the observation group was statistically different from the control group (*P* < 0.05).

In the observation group, the abscess was detected in 21 cases, fistula tract in 20 cases, and internal mouth in 23 cases; the detection accuracy was 84%, 80%, and 92%, respectively. In the control group, 15 cases of abscess, 17 cases of the fistula tract, and 18 cases of internal mouth were detected, with the detection accuracy of 60%, 68%, and 72% in turn. The detection rate of the observation group was statistically different from the rate of the control group (*P* < 0.05) (Figure 10).

## 4. Discussion

Infection, sex hormones, and immune factors are the important reasons for causing anal fistula with abscess. In the past, clinical diagnosis was made by combining clinical symptoms, anal digital examination, and colonoscopy, but the number of abscesses, lesions, and fistula tube walking conditions could not be determined, so it was necessary to use imaging data for diagnosis. MRI examination has a very high discrimination rate for all soft tissues, which can accurately reflect the perianal anatomical structure. Therefore, an MRI examination was carried out this time. In this research, the second to fifth convolutional blocks of the visual geometric group network were used to extract the depth features by transfer learning. Then, a deep learning model of multimode feature fusion was built, and the whole network was trained by maximizing the energy proportion of feature layers. It was compared with the FCN algorithm to analyze its related performance. It was found that the similarity coefficient (85.37%), accuracy (80.02%), and recall rate (79.38%) of the improved deep learning algorithm were significantly higher than the similarity coefficient (70.18%), accuracy (67.82%), and recall rate (66.92%) of the FCN algorithm (*P* < 0.05). With the increase of the number of convolutional layers, the segmentation accuracy of the CNN algorithm was also continuously improved. The similarity coefficient (88.82%), accuracy rate (85.19%), and recall rate (89.66%) of the 20-layer deep learning algorithm increased significantly in contrast to the similarity coefficient (47.62%), accuracy rate (49.88%), and recall rate (51.62%) of the 6-layer CNN (*P* < 0.05). The research findings of Panes et al. [18] also confirmed this conclusion. It was found that the MRI test method based on the deep learning algorithm of multimode feature fusion had a higher authenticity.

The algorithm was applied to the imaging diagnosis of perianal abscess patients, suggesting that there were 4 cases of intersphincter type, 10 cases of trans-sphincter type, 7 cases of suprasphincteric type, and 2 cases of extrasphincteric type in the observation group, with a detection rate of 84%; 3 cases of intersphincter type, 7 cases of trans-sphincter type, 5 cases of suprasphincter type, and 1 case of extrasphincteric shape were detected in the control group, and the detection rate was 64%. Reginelli et al. [19] showed that, after the use of a self-made adjustable anorectal water sac, image diagnosis with axial ligation-enhanced T1WI-SPIR sequence was found to be able to provide richer details of the branch and inner opening of the malfunction canal. The observation group had 21 cases of abscess, 20 cases of fistula position, and 23 cases of internal orifice position; there were 15 cases of abscess position, 17 cases of fistula position, and 18 cases of internal orifice position in the control group. The detection accuracy of abscess location (84%), impotent tract location (80%), and internal orifice location (92%) in patients from the observation group was significantly higher than that of abscess location (60%), impotent tract location (68%), and internal orifice location (72%) of the control group (*P* < 0.05). The research of Panes et al. [20] found that the experimental group carried out MRI diagnosis to detect 23% of the intersphincter type, 39% of the trans-sphincter type, 22% of the supersphincter type, and 15% of the extrasphincteric type, which was not statistically markedly different from the control group. Besides, 99% of perianal abscesses, 97% of fistulas, and 100% of internal mouth were detected, which were higher than routine preoperative examinations. Therefore, MRI had a high application value in improving the detection rate of anal fistulas and abscesses.

## 5. Conclusion

To explore the characteristic diagnosis and prognostic effects of perianal abscess, the deep learning algorithm based on multimodal feature fusion was applied to analyze and process the MRI images of patients with perianal abscesses. The results revealed that deep learning-based MRI image feature analysis of perianal abscess and fistula had high diagnostic accuracy and played a positive role in improving the detection rate of diseases, accuracy of disease classification, etc., which could be applied and promoted in clinical practice. However, there are still some shortcomings in this study. In this study, there are fewer groups in the experiment process. Therefore, the experimental results are subjective to a certain extent. There is also a small selection of research samples, and the test results are not suitable for large-scale use. The scope of research will be further expanded in future work.

## Data Availability

The data used to support the findings of this study are available from the corresponding author upon request.
